# Visual Performance of Eyes with Residual Refractive Errors after Implantation of an Extended Vision Intraocular Lens

**DOI:** 10.1155/2023/7701390

**Published:** 2023-05-04

**Authors:** Laureano A. Rementería-Capelo, Inés Contreras, Aida Morán, Pilar Lorente-Hevia, Laura Mariñas, Javier Ruiz-Alcocer

**Affiliations:** ^1^Clínica Rementería, Madrid, Spain; ^2^Hospital Universitario Ramón y Cajal, Madrid, Spain; ^3^Instituto Ramón y Cajal de Investigaciones Sanitarias (IRYCIS), Madrid, Spain; ^4^Optics and Optometry Department, Universidad Complutense de Madrid, Madrid, Spain; ^5^Clinical and Experimental Eye Research Group, Universidad Complutense de Madrid, UCM 971009, Madrid, Spain

## Abstract

**Background:**

To analyze the tolerance on distance vision of different combined residual astigmatic situations in patients implanted with a novel wavefront shaping extended depth of focus (EDoF) intraocular lens (IOL).

**Methods:**

The study included patients implanted with the Acrysof® IQ Vivity® IOL. Uncorrected (UDVA) and corrected distance visual acuity (CDVA) were measured three months after surgery, considering CDVA as the reference situation of the study. Distance VA was also measured in different refractive situations: (A) with 0.50 diopters (D) of positive (myopization) and negative (hyperopization) defocus and (B) with a residual mixed astigmatic refraction induced by adding a combination of −0.25 D spherical and 0.50 D cylindrical lenses placed in vertical (against the rule-ATR), oblique, and horizontal (with the rule-WTR) positions.

**Results:**

The study included 30 eyes of 30 patients. UDVA and CDVA were −0.04 ± 0.05 and −0.05 ± 0.05 logMAR, respectively. VA values with +0.50 D and −0.50 D of defocus were 0.01 ± 0.06 and 0.00 ± 0.04 logMAR, respectively. VA was better with distance correction (*p* < 0.001) and no differences were found between the myopic and the hyperopic situations (*p*=0.09). Distance VA for the ATR, oblique, and WTR astigmatic situations was 0.01 ± 0.05, 0.01 ± 0.06, and 0.01 ± 0.04 logMAR, respectively. VA was better for the reference situation (*p* < 0.001) and no differences were found among the three astigmatic situations (*p*=0.21).

**Conclusions:**

Low residual defocus and mixed astigmatic errors, regardless of its orientation, seem to be tolerated by patients implanted with the studied EDoF IOL. This trial is registered with NCT05392998. Registered 26 May 2022-Retrospectively registered.

## 1. Background

Multifocal intraocular lenses (IOLs) have been shown to be a good option for cataract surgeons aiming to offer improved vision at different distances. Trifocal IOLs provide enhanced vision at far, intermediate and near distances and have overcome the characteristic V-pattern with 2 peaks corresponding to near and far vision provided by bifocal IOLs [1]. Although multifocal IOLs could increase spectacle independence in patients undergoing cataract surgery, these lenses could also increase some visual disturbances such as glare or haloes [2].

“Extended depth of focus” (EDoF) IOLs are currently available for cataract surgeons. They employ different optical technologies to achieve extended vision [3], increasing the range of vision while minimizing visual disturbances induced by classic diffractive multifocal designs [3]. In fact, current clinical results show that EDoF lenses could increase the range of vision from far to intermediate distances and reduce the perception of visual disturbances of the patients [4].

In addition, while it has been reported that issues such as biomechanical properties of the cornea, inaccurate calculations, or misalignments of the IOLs may lead into residual refractive errors that will likely decrease the visual quality of patients with bifocal or trifocal IOLs [5], less is known about their impact on EDoF designs.

Therefore, regardless of the optical improvements achieved by EDoF lenses, better knowledge of the spherical and/or astigmatic tolerance of EDoF IOLs is crucial for an appropriate surgical planning and for avoiding postoperative refractive corrections. Thus, the aim of this study is to analyze the impact of mild amounts of residual spherical defocus and astigmatism in patients implanted with a novel EDoF IOL.

## 2. Patients and Methods

This prospective case series study was performed at Clínica Rementería, Madrid, Spain and included patients who had undergone bilateral implantation of a nondiffractive extended vision IOL. The study followed the tenets of the Declaration of Helsinki and was reviewed and approved by the Ethics Committee of the Hospital Clínico San Carlos, Madrid. Informed consent was obtained from all patients prior to inclusion.

Both for surgical and visual function procedures, we followed the methods employed in previous works developed in our clinic [6, 7]. The study included patients >40 years old that underwent routine cataract surgery and IOL implantation. Exclusion criteria included corneal astigmatism ≥1.0 diopters (D), amblyopia, previous ocular surgery, and presence of ocular pathologies and abnormal iris. Patients with intra- or postoperative complications, with a postoperative distance corrected visual acuity (DCVA) <20/20 and with postoperative refractive astigmatism >0.50 D were also excluded. Inclusion and exclusion criteria were assessed by an ophthalmologic examination including refraction, screening for ocular conditions and/or systemic diseases, biomicroscopy, and fundus examination.

### 2.1. Surgical Procedure

All cataract surgeries *were carried out by one experienced surgeon (L.A.R)* under topical and intracameral anesthesia, through a 2.2 mm clear-cornea incision at 135 degrees and “stop and chop” phacoemulsification [6, 7]. *Moreover, IOL implantation was guided by the VERION® System (Alcon Laboratories, Inc, Fort Worth, USA).*

### 2.2. Intraocular Lens

In this study, all patients were implanted with the AcrySof® IQ Vivity® *(Alcon Laboratories, Inc, Fort Worth, USA)*. This lens is presented as an extended range-of-vision IOL based on a wavefront-shaping technology called X-WAVE™. The lens has a central 2.2-mm optical zone, containing two nondiffractive transition elements changing the wavefront of the central light beams and focuses almost all the light from the IOL in that specific range [8]. The anterior surface of the IOL is designed with negative spherical aberration to compensate for the positive spherical aberration of the cornea [9].

The material of the lens is a hydrophobic acrylate/methacrylate copolymer with ultraviolet and blue light filters and a refraction index of 1.55. The optic diameter is 6 mm while the overall diameter is 13 mm. The lens is available is spherical powers from +15.0 D to +25.0 D [10].

### 2.3. Postoperative Clinical Assessment

Patients were evaluated one day, one week, one month, and three months postoperatively. Patients fulfilling inclusion criteria were approached to be included in the study at the three-month visit. Patients agreeing to participate underwent the explorations included in the study protocol.

Initially, uncorrected distance *(UDVA)*, intermediate *(UIVA)* (60 cm), and near *(UNVA)* (40 cm) visual acuity were measured with an ETDRS chart at 4 m, followed by subjective refraction. As in previous studies, once the best distance correction was obtained, further VA evaluation procedures were performed with the FrACT3.9.9a version of the Freiburg Acuity Test software package [6, 7, 11].

Once patients achieved the best distance correction (reference situation), a monocular analysis of VA at distance vision was conducted under different induced conditions. First, both a myopic and hyperopic defocus of **0.50** D were simulated. After these measurements, a mixed astigmatism with different orientation was also induced by adding a combination of −0.25 D spherical and 0.50 D cylindrical lenses placed in vertical (against the rule-ATR), oblique and horizontal (with the rule-WTR) positions.

### 2.4. Statistical Analysis

The calculation of the required sample size was based on monocular CDVA. A difference of 0.2 logMAR units was assumed to be clinically significant and a standard deviation of 0.05 was considered [12]. Based on this assumption and *α* of 0.05 and power of 0.8, it was calculated that 25 eyes were required.

Data analysis was performed using SPSS for Windows V.20.0 (*SPSS Inc, Chicago, USA*). The normal distribution of variables was assessed using the Kolmogorov–Smirnov test. A repeated-measures analysis of variance (ANOVA) was used to gauge any statistically significant difference within the different situations. Post-hoc multiple comparison testing was performed using the Holm–Sidak method. Differences were considered to be statistically significant when the *p* value was <0.05 (i.e., at the 5% level).

## 3. Results

A total of 30 eyes of 30 patients with a mean age of 69.37 ± 7.10 years were included in the study. Demographic preoperative data are shown in Table 1. Three months after the surgery, mean UDVA, UIVA, and UNVA were: −0.04 ± 0.06, 0.08 ± 0.07, and 0.25 ± 0.09 logMAR, respectively. The postoperative manifest refraction of the subjects showed a mean sphere of −0.02 ± 0.07 D (range −0.25 to 0.0 D), a mean cylinder of −0.04 ± 0.13 D (range −0.50 to 0.0 D), and a spherical equivalent of 0.02 ± 0.07 D (range −0.25 to 0.0 D).

**Figure 1:**
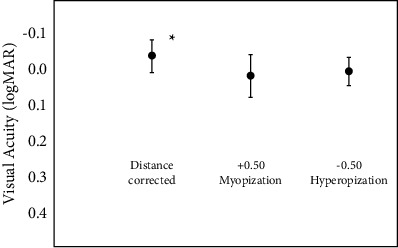
Mean logMAR distance corrected visual acuity (VA) (reference situation) and with ±0.50 diopters (D) induced defocus. ^*∗*^Represents statistically significant differences among the three situations analyzed.

Figure 1 shows the results of the group with the best distance correction and with ±0.50 D of defocus (myopization and hyperopization). With distance correction, VA was better if compared to both defocus situations (*p* < 0.001). However, this difference would not be clinically relevant. At the same time, no differences were found between the myopic and the hyperopic situations (*p*=0.09).

Besides, the proportion of patients in which VA remained stable or decreased significantly was also calculated. With residual myopic defocus, the proportion of patients in which VA remained unchanged or lost ≤1 lines of vision was 53%, while it was 67% for the hyperopic situation.

**Figure 2:**
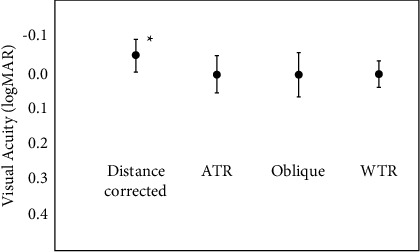
Mean logMAR distance corrected visual acuity (**CDVA**) (reference situation) and with a residual mixed astigmatic refraction induced by adding a combination of −0.25 diopter (D) spherical and 0.50 D cylindrical lenses placed in vertical (against the rule-ATR), oblique, and horizontal (with the rule-WTR) positions. ^*∗*^Represents statistically significant differences among the four situations analyzed.

The results of the induced astigmatic situations are presented in Figure 2. VA for the reference distance corrected situation was better than for the astigmatic situations (*p* < 0.001), although again this difference would not be clinically relevant VA for the ATR, oblique and WTR astigmatism showed no statistically significant differences (*p*=0.21).

For the astigmatic situations, the proportion of patients in which VA remained stable or lost ≤1 lines of vision was also calculated. For the ATR, oblique, and WTR residual astigmatism, 60%, 70%, and 60% of patients, respectively, showed stable VA values.

We did not record any adverse event in any visit or during the development of the study.

## 4. Discussion

EDoF lenses have been developed to offer functional vision at different distances and to overcome some limitations related to multifocal (bifocal or trifocal) IOLs such as postoperative dysphotopsias. However, residual refractive errors may minimize the potential optical characteristics of these novel designs. In addition, considering that a significant proportion of patients present preoperative astigmatism, the aim of this study is to analyze whether low residual spherical defocus of different signs or astigmatism at different orientations have an impact on distance VA after implantation of a novel EDoF IOL.

As regards residual spherical defocus, although it leads to a statistically decrease in VA this would have no clinical impact. In fact, while mean CDVA was −0.05 logMAR (around 1.1 decimal), mean VA with simulated myopia and hyperopia was 0.01 and 0.00 logMAR, respectively (around 1.0 decimal) decimal. The impact of defocus errors in patients with trifocal designs has also been reported [5, 7], with a greater deterioration of distance visual acuity with defocus. Therefore, our results suggest that these EDoF designs might better tolerate certain levels of defocus compared to diffractive trifocal lenses. Nevertheless, besides distance VA, it should be considered that defocus errors also change intermediate and near distances of vision with multifocal IOLs which may induce and additional impact to an optimal visual performance with these designs.

The proportion of patients in which VA remained unchanged or lost ≤ 1 line of vision was high, of 53% with induced myopia and 67% with induced hyperopia. These results suggest that a high proportion of patients will tolerate low spherical refractive errors, but clinicians should of those patients that might complain due to a potential VA decrease after surgery. At the same time, uncorrected residual refractive errors may lead to increased halo, which could also be bothersome to some patients.

Residual mixed astigmatism at different orientations was also simulated and compared to the reference situation. Again, although there was a decrease in VA, it remained ≥1.0 decimal for all situations, so that the clinical impact of the induced astigmatism could be considered as minimal. Previous studies suggested that ATR astigmatisms may be less tolerated than oblique and WTR astigmatisms [6, 13]. No direct comparisons can be made with those studies because the current study analyzed a low mixed astigmatism while the others analyzed low myopic astigmatism with trifocal IOLs [6] or astigmatisms with monofocal IOLs [13]. However, our results suggest that low mixed astigmatisms with the EDoF lens under study are well tolerated regardless of their orientation.

In this case, the proportion of patients that maintained a stable VA with induced residual astigmatism was approximately 60% for the three orientations. The results were similar to those obtained for the myopic and the hyperopic situations. Therefore, it seems that averaged values showed a minimal impact but complaints due to VA deterioration should be carefully assessed since not all patients showed the same tolerance to residual refractive errors.

It should be noted that, to strengthen potential conclusions raised from these results, future studies should be performed with a larger sample and under different conditions. At the same time, this study simulated certain spherical and astigmatic residual refractive errors; nevertheless, future studies with different refractive combinations will be of great interest.

*In addition,* to be included in this study, patients met strict inclusion criteria. For example, no patients with successful preoperative refractive surgery were included and UDVA was at least 1.0 decimal for all patients. The combination of different ocular aberrations (i.e., due to previous myopic or hyperopic corneal ablations) with the IOL may offer significantly different outcomes [14]. At the same time, the study assessed objective VA results, but different profiles of patients could show different levels of (objective or subjective) tolerance to low refractive errors. Thus, similar studies should be performed to analyze the impact of residual refractive errors in groups of patients representing a wider proportion of the population that will undergo cataract surgery.

To the best of our knowledge, this is the first study that analyzed the impact of spherical and astigmatic errors in patients with this novel nondiffractive EDoF. Different optical designs such as small aperture, bioanalogic, and diffractive optics [3] have been developed to create an elongated focus that improves the range of vision. Thus, the impact of residual refractive errors should be also assessed in patients with different EDoF designs.

In conclusion, the results of this study suggest that low levels of residual myopia, hyperopia, and mixed astigmatisms, regardless of their orientation, seem to be tolerated at distance vision in patients with the IOL under study. Nevertheless, residual refractive errors should be minimized in patients with premium lenses since some patients may experience a greater loss in visual acuity with low refractive errors.

## Figures and Tables

**Table 1:** Preoperative characteristics of the eyes included in the study. Values provided are mean ± standard deviation.

Number of eyes	Photopic pupil (mm)	IOL power (D)	Sphere (D)	Cylinder (D)	Spherical equivalent (D)	CDVA (LogMAR)
30	2.66 ± 0.46	21.70 ± 2.25	0.34 ± 1.73	−0.34 ± 0.38	0.01 ± 0.93	0.17 ± 0.23

CDVA corrected distance visual acuity.

## Data Availability

The datasets generated and/or analysed during the current study are not publicly available but are available from the corresponding author upon reasonable request.
